# Ptc6 Is Required for Proper Rapamycin-Induced Down-Regulation of the Genes Coding for Ribosomal and rRNA Processing Proteins in *S. cerevisiae*

**DOI:** 10.1371/journal.pone.0064470

**Published:** 2013-05-21

**Authors:** Asier González, Carlos Casado, Joaquín Ariño, Antonio Casamayor

**Affiliations:** Institut de Biotecnologia i Biomedicina (IBB) and Departament de Bioquímica i Biologia Molecular, Facultat de Veterinària, Universitat Autònoma de Barcelona, Cerdanyola del Vallès, Spain; University of Leuven (KU Leuven), Faculty of Medicine, Belgium

## Abstract

Ptc6 is one of the seven components (Ptc1-Ptc7) of the protein phosphatase 2C family in the yeast *Saccharomyces cerevisiae*. In contrast to other type 2C phosphatases, the cellular role of this isoform is poorly understood. We present here a comprehensive characterization of this gene product. Cells lacking Ptc6 are sensitive to zinc ions, and somewhat tolerant to cell-wall damaging agents and to Li^+^. Ptc6 mutants are sensitive to rapamycin, albeit to lesser extent than *ptc1* cells. This phenotype is not rescued by overexpression of *PTC1* and mutation of *ptc6* does not reproduce the characteristic genetic interactions of the *ptc1* mutation with components of the TOR pathway, thus suggesting different cellular roles for both isoforms. We show here that the rapamycin-sensitive phenotype of *ptc6* cells is unrelated to the reported role of Pt6 in controlling pyruvate dehydrogenase activity. Lack of Ptc6 results in substantial attenuation of the transcriptional response to rapamycin, particularly in the subset of repressed genes encoding ribosomal proteins or involved in rRNA processing. In contrast, repressed genes involved in translation are Ptc6-independent. These effects cannot be attributed to the regulation of the Sch9 kinase, but they could involve modulation of the binding of the Ifh1 co-activator to specific gene promoters.

## Introduction

Type 2C Ser/Thr protein phosphatases (PP2Cs) are a group of monomeric enzymes highly conserved throughout evolution. The classification of these proteins according to their primary structure shows that in fungi there are five major groups of PP2Cs [1]. In the budding yeast *Saccharomyces cerevisiae* the PP2C family is composed of seven members (Ptc1-7) that include representatives of all structural groups previously described. The last member incorporated to the Ptc family was *YCR079w* (*PTC6*), which was presumed for many years to encode a type 2C enzyme [2], [3], but whose phosphatase activity was only recently demonstrated [4]. It is known that the *PTC7* gene can produce 2 different polypeptides by differential splicing [5]. As occurs in higher eukaryotes, yeast PP2Cs were initially associated to the regulation of cell growth and stress signaling. Our current knowledge, however, suggests that PP2C functions are much more diverse (for a review see [1] and references therein). While the subcellular localization of Ptc1-4 is cytoplasmatic or nuclear [6], [7], Ptc5, Ptc6 and the spliced version of Ptc7 are located in the mitochondria [5], [8]–[11]. There is some controversy, however, about the localization of Ptc6 within this organelle because it has been proposed that it is localized to either the mitochondrial intermembrane space or the mitochondrial matrix [11], [12].

In spite of the growing body of knowledge, our understanding of the function(s) and regulatory mechanisms for each specific PP2C isoform is still rather limited, and this is particularly true for the mitochondrially-located isoforms. For instance, only one cellular target for Ptc6 has been described so far. Both, Ptc6 and Ptc5, seem to dephosphorylate Ser313 of Pda1, a component of the E1α subunit of the pyruvate dehydrogenase (PDH) complex that catalyzes the oxidative decarboxylation of pyruvate to form acetyl-CoA thus connecting glycolysis and the tricarboxylic acid cycle [13]. In fact, the PDH complex activity in *ptc6* or *ptc5* strains is greatly reduced [10], [13]. Moreover, *ptc6* mutants are unable to degrade aconitase in a Pep4-dependent fashion and have impaired mitochondrial transport into the vacuole after prolonged stationary phase, suggesting that Ptc6 plays a role in the mitochondrial degradation process known as mitophagy (see [14] and references therein). As a consequence, Ptc6 is necessary for survival of lactate-growing stationary phase cells [11]. The role of Ptc6 in mitophagy is probably exerted through Rtg3, the transcription factor that mediates the RTG (*r*e*t*ro*g*rade signaling pathway) response [15]. Due to this function in mitophagy, Ptc6 has received the Aup1 (for *au*tophagy-related protein *p*hosphatase) alias [11].

Remarkably, among the *ptc* family mutants, only *ptc1* and *ptc6* are sensitive to rapamycin (an inhibitor of the activity of Tor kinases) and caffeine (a compound that has been related with the cell wall integrity pathway and that can also act as an inhibitor of Tor kinases) [4], [16]–[19]. On the other hand, overexpression of *PTC6* renders cells tolerant to rapamycin [4]. The TOR (*t*arget *o*f *r*apamycin) pathway is a conserved signaling network (for a review, see [20] and references therein) important for cell growth control that involves the phosphatidylinositol kinase-related protein kinase (PIKK) Tor1 and Tor2. These kinases are found in two functionally and structurally distinct multiprotein complexes: TORC1 and TORC2, each of which signals through a different set of effector pathways. TORC1 is rapamycin-sensitive, whereas TORC2 is rapamycin insensitive [21]. TORC1 activates cell growth by positively regulating diverse anabolic processes, such as transcription, protein synthesis, ribosome biogenesis, nutrient transport, and mitochondrial metabolism, whereas it represses several catabolic pathways, such as mRNA degradation, ubiquitin-dependent proteolysis, autophagy and apoptosis. However, the molecular mechanisms by which TORC1 signals to these diverse processes in both yeast and mammals are still open to discussion. In particular, only a few substrates of either TORC1 or its direct effectors such as the AGC kinase Sch9 [22] or the Tap42/Tip41-PP2A/Sit4 system (see [20] and references therein) are known.

The rapamycin-sensitive phenotype of the *ptc1* mutant lead us to recently uncover a role for Ptc1 in normal signaling through the TORC1 pathway, possibly by regulating a step upstream of Sit4/Tip41 function [19]. Consequently, we also decided to investigate the nature of the possible functional connection between Ptc6 and this pathway. Our results suggest that the role of Ptc1 and Ptc6 in maintaining the normal function of TORC1 pathway is different. Genetic studies also indicate that the rapamycin-sensitive phenotype derived by the lack of Ptc6 is not mediated by its role on the regulation of the PDH complex or the mitophagy process. Remarkably, transcriptomic analyses show that mutation of *PTC6* significantly attenuates the transcriptional changes caused by rapamycin, mainly at the level of repressed genes. In this study we propose that the inability to repress transcription of certain genes in response to rapamycin may be the cause of the phenotypes observed in Ptc6-deficient cells.

## Materials and Methods

### Yeast and *Escherichia coli* growth conditions

Yeast cells were incubated at 28°C in YPD medium (1% yeast extract, 2% peptone and 2% glucose) or in synthetic medium [23] containing 2% glucose and lacking the appropriate selection requirements. The Low Ammonium medium is synthetic medium containing 2% glucose and supplemented with 10 mM ammonium sulphate, 40 mg/l methionine, 20 mg/l histidine and 100 mg/l leucine.

*E. coli* DH5α cells were used as plasmid DNA host and were grown at 37°C in LB broth supplemented with 50 µg/ml ampicillin, when required. Bacterial and yeast cells were transformed using standard methods. Standard recombinant DNA techniques were performed as described elsewhere.

The sensitivity of yeast cells to diverse stressing agents was evaluated by growth on agar plates (drop tests) as described in [24]. Sensitivity of each strain to rapamycin was evaluated in liquid cultures as previously described [25] and represented as relative growth respect the untreated strain.

### Gene disruptions and plasmid construction

The *S. cerevisiae* strains used in this work are listed in the Table 1. Single *kanMX* deletion mutants in the BY4741 background were generated in the context of the *Saccharomyces* Genome Deletion Project [26]. Replacement of the *PTC6* coding region by the *nat1* marker from *Streptomyces noursei* was accomplished as follows: the 1.40 kbp DNA fragment containing the *nat1* gene, flanked by genomic sequences corresponding to −40/+5 and +1310/+1352 relative to the *PTC6* ATG codon, was amplified from the plasmid pAG25 [27] with the oligonucleotides 5′-PTC6-disr_nat and 3′-PTC6-disr_nat (Table S1). The *ptc6::nat1* disruption cassette was transformed in the appropriate strains and positive clones were selected in the presence of 100 µg/ml nourseothricin (Werner BioAgents). Double mutants *ptc2 ptc6* and *ptc3 ptc6* were constructed by introducing the *ptc6::nat1* disruption cassette in the *ptc2:kanMX* and *ptc3::kanMX* mutants from the BY4741 deletion bank.

**Table 1:** Yeast strains used in this work.

Name	Relevant genotype	Source/Reference
BY4741	*MATa his3Δ1 leu2Δ met15Δ ura3Δ*	[26]
AGS71	BY4741 *ptc6::nat1*	This work
MAR14	BY4741 *ptc1::nat1*	[52]
CCV190	BY4741 *ptc2::kanMX4 ptc6::nat1*	This work
CCV191	BY4741 *ptc3::kanMX4 ptc6::nat1*	This work
AGS60	BY4741 *ptc6::kanMX4 ptc1::nat1*	This work
CCV22	BY4741 *ure2::kanMX4 ptc6::nat1*	This work
CCV24	BY4741 *gat1::kanMX4 ptc6::nat1*	This work
AGS72	BY4741 *tor1::kanMX4 ptc6::nat1*	This work
AGS73	BY4741 *tip41::kanMX4 ptc6::nat1*	This work
AGS74	BY4741 *sit4::kanMX4 ptc6::nat1*	This work
CCV25	BY4741 *ptc5::kanMX4 ptc6::nat1*	This work
CCV26	BY4741 *lpd1::kanMX4 ptc6: nat1*	This work
AGS75	BY4741 *pda2::kanMX4 ptc6::nat1*	This work
AGS76	BY4741 *pkp1::kanMX4 ptc6::nat1*	This work
AGS77	BY4741 *pdb1::kanMX4 ptc6::nat1*	This work
CCV29	BY4741 *pep4::kanMX4 ptc6::nat1*	This work
CCV30	BY4741 *atg1::kanMX4 ptc6::nat1*	This work
YVM70	W303-1A Ifh1-myc^13^	[45]
AGS82	W303-1A Ifh1-myc^13^ *ptc6::nat1*	This work

The single *kanMX* deletion mutants in the BY4741 background that were generated in the context of the *Saccharomyces* Genome Deletion Project are not listed here.

The plasmids containing *LacZ* translational fusions with the promoters of *GAP1*, *GLN1, GDH1* and *MEP1* have been previously described in [19]. The construction of plasmids YEp195-*PTC1* and YEp195-*PTC1* ^[D58N]^ was reported earlier in [28] and [19], respectively. Plasmid pJU676 (pRS416-SCH9-5HA) was a generous gift from R. Loewith (Department of Molecular Biology Sciences. University of Geneva. Switzerland).

### RNA purification, cDNA synthesis and DNA microarray analysis

For RNA purification, 30 ml of yeast cultures of wild type BY4741 and its derivatives mutant strains were grown at 28°C in YPD medium until *A*_660_ 0.6–0.8 and, when required, treated with 200 ng/ml rapamycin or drug vehicle alone (90% ethanol and 10% Tween P20) for 1 hour. Cells were harvested by centrifugation and washed with cold water. Dried pellets were kept at −80°C until RNA purification. Total RNA was extracted using the RiboPure-Yeast kit (Ambion) following the manufacturer's instructions. RNA quality was assessed by electrophoresis in denaturing 0.8% agarose gel and quantified by measuring *A*_260_ in a BioPhotometer (Eppendorf). Transcriptional analyses using DNA microarrays developed in our laboratory were performed exactly as described in [29]. Briefly, 8 µg of total RNA for each sample were employed for the cDNA synthesis and labeling using the indirect labeling kit (CyScribe Post-Labeling kit, GE-Amersham Biosciences) in conjunction with Cy3-dUTP and Cy5-dUTP fluorescent nucleotides. The cDNA obtained was dried and resuspended in the hybridization buffer. The amount of DNA and the labeling efficiency was evaluated with a Nanodrop spectrophotometer (Nanodrop Technologies). Fluorescently labeled cDNAs were combined and hybridized to yeast genomic microchips constructed in our laboratory by arraying 6014 different PCR-amplified open reading frames (ORFs) from *S. cerevisiae* [30], [31]. Pre-hybridization, hybridization and washing conditions were essentially as described previously [32]. The slides were scanned with a ScanArray 4000 apparatus (Packard BioChips Technologies), and the output was analyzed using GenePix Pro 6.0 software. Spots with either a diameter smaller than 120 µm or fluorescence intensities for Cy3 (indocarbocyanine) and Cy5 (indodicarbocyanine) lower than 150 units, were not considered for further analysis.

Three different sets of microarray experiments were performed. In the first set of experiments we compared the expression profiles of *ptc6* mutant cells with that of wild type cells by performing two independent experiments (biological replicates), each in duplicate (dyes were swapped to avoid dye-specific bias). In the second series of experiments, we compared the transcriptomic profiles of *ptc1 ptc6* double mutant cells with that of wild type cells. Two independent experiments were performed, each in duplicate. For these two set of experiments we only considered for further analysis genes with data in at least two out four spots. In the last set of experiments we compared the transcriptomic profiles of WT, *ptc6*, and *ptc1ptc6* cells in the presence and the absence of rapamycin. In this case, data from duplicate experiments were combined, and the mean was calculated. A given gene was considered to be induced or repressed when the expression ratio was higher than 2.0 or lower than 0.50, respectively. The GEPAS3.0 software, now implemented in the Babelomics tool (http://babelomics.bioinfo.cipf.es/), was used to pre-process the data [33]. The MIPS Functional Catalogue Database [34], at http://mips.helmholtz-muenchen.de/proj/funcatDB/search_main_frame.html, and Gene Ontology Enrichment tool available at YeastMine [35] (http://yeastmine.yeastgenome.org), were used for the functional distribution of gene lists.

Different levels of dependence on Ptc6 were defined as “totally dependent” (TD), “strongly dependent” (SD), “weakly dependent” (WD) and independent, according to the expression of up- or down-regulated genes after rapamycin treatment in *ptc6* cells in comparison with wild type cells, as previously reported [36]. The data discussed in this publication have been deposited in NCBI's Gene Expression Omnibus database [37] and are accessible through GEO SuperSeries accession number GSE38260.

### Chromatin immunoprecipitation assays

Chromatin cross-linking and immunoprecipitation were carried out based on previously described methods [38] with several modifications. Forty ml cultures were grown up to OD_660_ 0.6–0.8 on YPD medium, and cells were exposed to 200 ng/ml rapamycin for 5, 15, 30 and 45 min. Then, cells were treated with 1.1 ml of 37% formaldehyde (1% final concentration) for 15 min at 24°C and quenched by addition of 2 ml of 2.5 M glycine for 5 min at 24°C. Cells were collected by centrifugation and washed twice with 10 ml of ice-cold HBS (50 mM HEPES-KOH, pH 7.5, 140 mM NaCl) and once with 1.5 ml of Lysis buffer (50 mM HEPES-KOH, pH 7.5, 140 mM NaCl, 1 mM EDTA, 1% Nonidet P 40, 0.1% sodium deoxycholate). The pellet was resuspended in 300 µl of Lysis buffer with 1 mM phenylmethylsulfonyl fluoride (PMSF) and complete protease inhibitor mixture (Roche Applied Science). One volume of zirconia-silica 0.5 mm beads (BioSpec) was added and cells were broken at 4°C by vigorous shaking (5 times for 25 s each at setting 5.5, with intervals of 1 min on ice) in a Fast Prep cell breaker (FastPrep 24, MP Biomedicals). The chromatin was sheared using a Bioruptor Plus UCD-300 sonication device (Diagenode) (high intensity, ten cycles of 30 s sonication interspersed with 60 s pause). The cleared lysate (whole cell extract, WCE) was recovered by centrifugation at 9300×*g* for 5 min at 4°C. For chromatin immunoprecipitation, 50 µl of Protein G-Sepharose (GE Healthcare) was coupled to 2.5 µg of anti c-myc monoclonal antibody (Covance). The anti-myc-Protein G-Sepharose complexes were incubated overnight with 200 µl of WCE (4 mg) at 4°C in a rotator. Sepharose-protein complexes were transferred to 96-well filter plates (MultiScreen, Millipore) and extensively washed at 4°C with 250 µl of the indicated solutions as follows: Four times with lysis buffer for 1 min, four times with lysis buffer containing 500 mM NaCl, twice with washing buffer (10 mM Tris-HCl, pH 7.5, 1 mM EDTA, 250 mM LiCl, 0.5% Nonidet P-40, 0.5% sodium deoxycholate), and twice with TE (10 mM Tris-HCl, pH 7.5, 1 mM EDTA). Washes were discarded by centrifugation at 180×*g*. Protein-DNA complexes were recovered from beads by incubation with 80 µl of elution buffer (50 mM Tris-HCl, pH 8.0, 10 mM EDTA, 1% SDS) at 65°C for 20 min. The supernatant was removed (60 µl), 240 µl of elution buffer were added, and samples were incubated overnight at 65°C. WCE controls were prepared from untagged cells by mixing 10 µl of WCE with 240 µl of elution buffer and incubating overnight at 65°C. Formaldehyde cross-links were reversed by incubation with 150 µg of proteinase K for 1 h at 37°C. The eluted DNA was purified with phenol-chloroform, precipitated with isopropanol, and dissolved in 30 µl (immunoprecipitated samples) or 50 µl of TE (WCE samples), and stored at −20°C. For PCR assays, 30 ng of the immunoprecipitated DNA was used. Oligonucleotides for PCR were designed to amplify 50- to 100-bp fragments (Table S1).

### Western blot and chemical fragmentation analysis

The Sch9 detection experiments were performed essentially as described in [22]. Briefly, yeast strains were grown on YPD to OD_660_ 0.5–0.6 at 28°C. Nine ml of cultures were mixed with TCA (final concentration 6%) and put on ice for at least 10 min before cells were pelleted at 1680×*g* for 2 min. The pellet was washed twice with 1 ml cold acetone and dried at 37°C. One-hundred µl of urea buffer (50 mM Tris pH 7.5, 5 mM EDTA, 6 M urea, 1% SDS, 1 mM PMSF) was added to the pellet and cell lysis was performed with zirconia-silica 0.5 mm beads (BioSpec) by vigorous shaking in a Fast Prep cell breaker (FastPrep 24, MP Biomedicals) as described above. Then, samples were heated for 10 min at 65°C. For NTCB (2-nitro-5-thiocyanatobenzoic acid) cleavage, 30 µl of 0.5 M CHES (pH 10.5) and 20 µl of NTCB (7.5 mM in H_2_O) were added and samples were incubated overnight at 24°C. Then, one volume of 2× sample buffer was added. Samples were fractionated by SDS-PAGE in 7.5% polyacrylamide gels and transferred to Immobilon PVDF membranes (Millipore). Membranes were incubated overnight with anti-HA antibody (Covance) at 1∶2000 dilution followed by the secondary HRP-conjugated anti-mouse IgG antibody (GE Healthcare) at 1∶20000 dilution. The immunocomplexes were visualized using ECL Western blotting detection kit (GE Healthcare). Chemiluminescence was detected using a LAS-3000 equipment (Fuji).

For Aco1 detection, whole cell lysates (10 ml of culture) were prepared by resuspending the cells in 200 µl of extraction buffer (50 mM Tris-HCl pH 7.5, 150 mM NaCl, 0.1% Triton X-100, 1 mM dithiothreitol, 10% glycerol, 2 mM phenylmethylsulfonyl fluoride and Complete inhibitor mixture (Roche Applied Science)). One volume of zirconia-silica 0.5 mm beads (BioSpec) was added and cells were broken at 4°C by vigorous shaking as above. After sedimentation at 500×*g* for 10 min at 4°C, the cleared lysate was recovered and the protein concentration was determined by Bradford assay. Total proteins (40 µg) were fractionated by SDS-PAGE in 10% polyacrylamide gels and transferred to Immobilon PVDF membranes (Millipore). Membranes were incubated overnight with anti-Aco1 antibody (a generous gift of Dr. S. Atrián, Universitat de Barcelona) at 1∶10000 dilution followed by the secondary HRP-conjugated anti-rabbit IgG antibody (GE Healthcare) at 1∶20000 dilution. The immunocomplexes were visualized using ECL Western blotting detection kit (GE Healthcare). Chemiluminescence was detected using a LAS-3000 equipment (Fuji).

### Other techniques

Total RNA was prepared as described above. Semi-quantitative RT-PCRs were performed using 100 ng of total RNA (except for *MEP1* amplifications, where 200 ng were used) and the Ready-To-Go RT-PCR Beads kit (GE Healthcare). Specific pairs of oligonucleotides were used (Table S1) to determine the levels of *GAP1* and *ACT1* (25 amplification cycles) and *MEP1* (30 amplification cycles). The PCR products were visualized in 2% agarose gels.

For quantitative RT-PCR, cDNA was synthesized from 1.5 µg of each RNA with the Superscript III First-Strand Synthesis System (Life Technologies). Five ng of each cDNA were used as a template, together with the pairs of oligonucleotides specified in the Table S1 and the Power SYBR green PCR Master mix (Applied Biosystems). Amplification reactions were carried out in a StepOnePlus Real-Time PCR System (Applied Biosystems). Fold-changes were calculated using the 2^−ΔCt^ method.

Evaluation of the promoter activity of diverse NCR-sensitive genes in response to rapamycin or to Low Ammonium Medium was performed using LacZ-reporters as described in [19].

Vacuolar staining and visualization was carried out using the lipophilic styryl dye FM4-64 (Molecular Probes) as described in [39].

Msn2 subcellular localization was performed using the pMSN2-GFP plasmid (a generous gift of F. Estruch, University of Valencia) as described in [19].

## Results

### Functional characterization of the *ptc6* mutant

**Figure 1:**
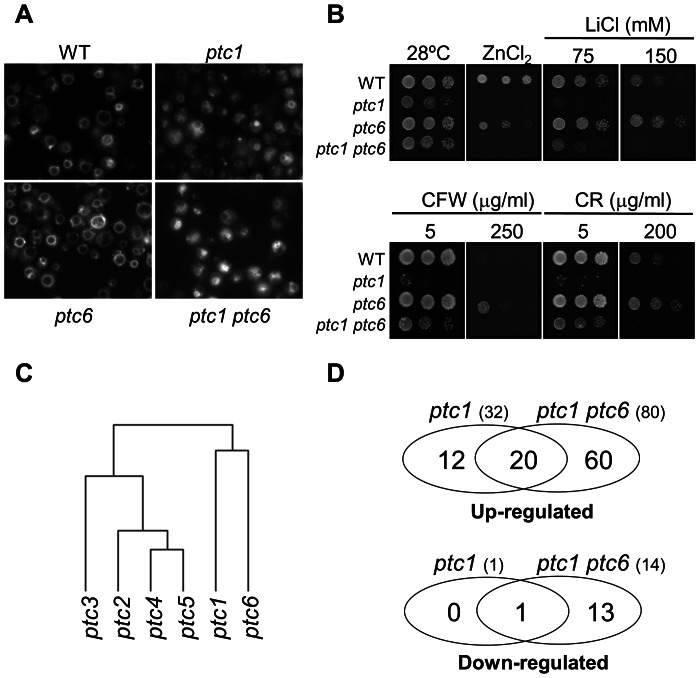
Characterization of *ptc6* mutant cells. A) Vacuole morphology is not altered in *ptc6* cells. Representative microscopic fields of wild type BY4741 (WT), *ptc1*, *ptc6* and *ptc1 ptc6* cells labeled with the lipophilic fluorescent dye FM4-64 to visualize vacuole morphology (×1000). B) Sensitivity of *ptc6* mutants to different stressing conditions. 1∶5 serial dilutions containing the same amount of cells of the strains above were spotted on YPD plates containing 6 mM ZnCl_2_, or the indicated concentrations of LiCl, Calcofuor White (CFW) or Congo Red (CR). Growth was monitored after 3 days, except for the plate of 150 mM LiCl (4 days). C) Clustering of the global expression profiles from mutants in each of the analyzed Ptc mutants. The dendrogram is based on the expression profiles previously obtained for each *PTC* mutant [29] with respect to the wild type strain and has been obtained using the Cluster Server (unweighted pair group method using arithmetic averages and correlation distance) at GEPAS [33]. D) Venn diagram showing the number of genes whose expression was considered to be induced (top) or repressed (bottom) in both *ptc1* and *ptc1 ptc6* double mutant cells exponentially growing on YPD. Data for the *ptc1* strain was taken from [29].

Because of the striking rapamycin-sensitive phenotype of *ptc6* cells, we considered necessary to carry out a comprehensive analysis of phenotypes derived from this mutation, and compare the phenotypes with those of the *ptc1* mutant, the only other *ptc* mutant that has been reported as sensitive to rapamycin [19]. We have found that cells lacking *ptc6*, in contrast to *ptc1* mutants, are not sensitive to high temperature (37°C), alkaline pH, or high amounts of calcium, copper or iron (not shown) and can grow on glycerol and ethanol as carbon sources (not shown). Similarly, the vacuolar structure of *ptc6* cells appears to be normal, whereas it is highly fragmented in the *ptc1* mutant (Figure 1A). The *ptc6* mutation renders cells slightly sensitive to zinc, albeit to less extent than that of *PTC1*. Remarkably, the *ptc6* mutant is somewhat tolerant to cell-wall damaging agents such as calcofluor white or Congo Red, as well as to Li^+^ ions (Figure 1B) and, in fact, lack of Ptc6 improves to some extent the deficient growth caused by the *ptc1* mutation under these conditions.

To gain insight into the possible cellular roles of Ptc6, we investigated by microchip analysis the changes in the global transcriptional profile caused by the *ptc6* mutation in cells growing under standard conditions. Disappointingly, we observe that lack of *PTC6* had almost no effect on the expression profile. The level of mRNA of only two genes, excluding *PTC6* itself, was found decreased (*FIT3* and *TIS11*) and, although mRNA levels of several genes involved in the lysine biosynthesis (*LYS1*, *LYS20*, *LYS21*, *LYS12* and *LYS9*) were slightly increased, they did not reach the defined threshold (2-fold increase). In any case, when we compared the reported expression profile of the *ptc1-5* mutants [29], obtained using the same DNA microarray platform and yeast genetic background, together with that of *ptc6* cells, it became evident that the expression profile of *ptc6* cells is quite different of that obtained for the *ptc1-5* mutants (Fig. 1C). We also evaluated the expression profile of *ptc1 ptc6* cells. Simultaneous deletion of *PTC1* and *PTC6* genes provoked more transcriptional changes than those observed previously for the single deletion of the *PTC1* gene (Fig. 1D). We found that the levels of mRNA for 80 genes were upregulated, whereas 14 genes, including *PTC1* and *PTC6*, were down-regulated in *ptc1 ptc6* double mutant cells, when compared to the wild type strain (see Tables S2 and S3 for the complete list of genes).

A substantial number of genes considered up-regulated in *ptc1 ptc6* cells (30 genes or 37.5% of the induced) were either related to the cell wall function or were found induced by cell-wall stress [40], as previously described for the *ptc1* mutant [29]. In fact, 18 out 20 genes whose expression was up-regulated at least two-fold in *ptc1* mutant cells [29] with valid data for the *ptc1 ptc6* mutant, were also up-regulated in *ptc1 ptc6* cells. The expression of the two other genes was either found consistently elevated (*NCA3*) or not induced at all (*PHO89*) in *ptc1 ptc6* cells.

Changes in the expression levels were quantitatively higher in the *ptc1 ptc6* double mutant than in *ptc1* cells. The average of the values of induction (in log_2_) for the set of 18 genes found simultaneously up-regulated (more than 2-fold) in both strains was higher in *ptc1 ptc6* (1.93, equivalent to 3.82-fold) than in *ptc1* cells (1.45, equivalent to 2.74-fold). This is also true when only the set of genes involved in cell wall integrity was considered.

Among the 60 genes specifically induced in *ptc1 ptc6* but not in *ptc1* or *ptc6* cells it is worth noting the presence of a set of 17 genes involved in carbon-compound and carbohydrate metabolism (p-value: 3.88E-06), six of them (*TSL3*, *GLC3*, *GIP2*, *GSC2*, *PGM2* and *GDB1*) were involved in the metabolism of energy reserves (p-value: 1.51E-05). We also found 7 genes (*ARN2*, *PDR5*, *FIT3*, *MEP2*, *ARN1*, *FIT1* and *FIT2*) involved in ion transport (p-value: 5.71E-04).

Taken together, the comparison of the phenotypic and transcriptomic data presented here, as well as the previously reported results [29] suggest that the cellular functions of Ptc6 are substantially different from those of Ptc1 and also from Ptc2-Ptc5.

### Ptc6 and Ptc1 participate in the TOR signaling pathway by different mechanisms

**Figure 2:**
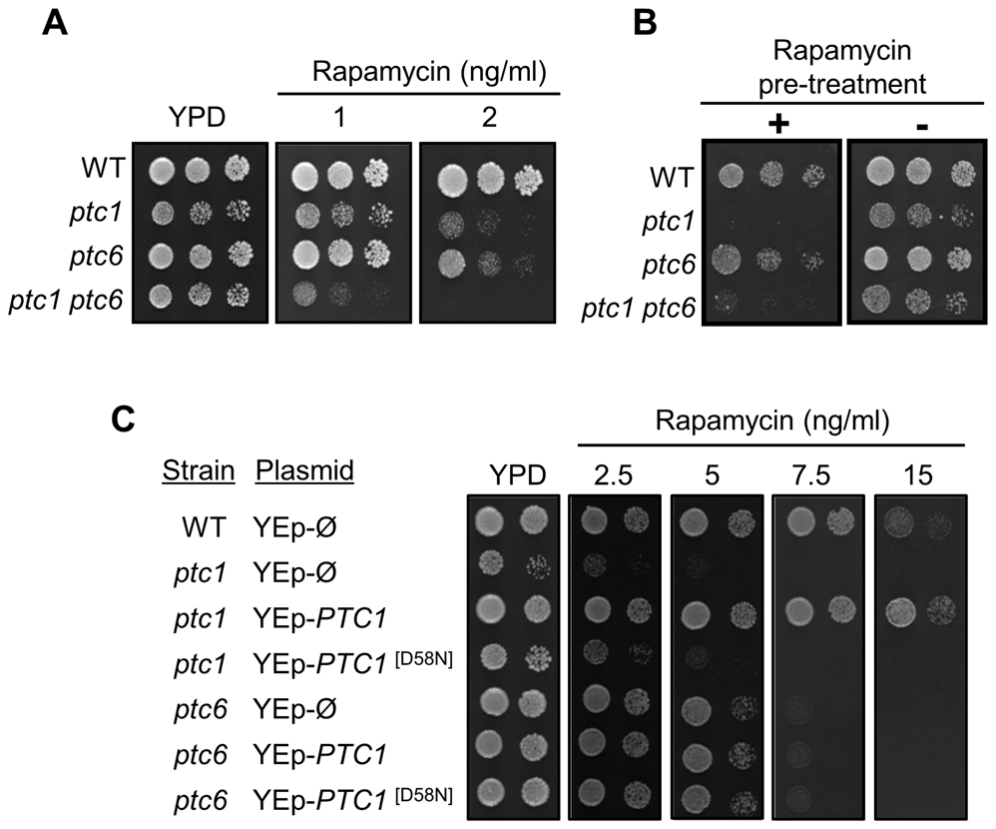
Ptc1 and Ptc6 differentially regulate the TOR signaling pathway. A) Wild-type strain BY4741 and the indicated mutants were spotted onto YPD plates containing the specified concentrations of rapamycin. Growth was monitored after 3 days of incubation at 28°C. B) Exponentially growing BY4741 cells and the indicated mutants at a OD_600_ of 0.8–1.0 growing in YPD medium were incubated either with 200 ng/ml rapamycin (+) or the vehicle (−), for 6 h. Cells were then washed twice, resuspended in YPD, spotted onto YPD plates and the growth monitored after 3 days. C) The BY4741 strain and its *ptc1* and *ptc6* derivatives were transformed with an empty high-copy-number YEplac195 vector (YEp-Ø) or with the same vector bearing either the wild type or a catalytically inactive form of Ptc1 (PTC1^[D58N]^). Cells were spotted as described above and incubated for 3 days.

As previously described, the *ptc1* strain is more sensitive to rapamycin than *ptc6* cells. We show here that the double mutant *ptc1 ptc6* was even more sensitive to this compound than *ptc1* cells (Figure 2A). Interestingly, the rapamycin-sensitive phenotype of *ptc1* is not altered at all by further deletion of *PTC2* or *PTC3*, which encode the closest structural relatives to Ptc1 (data not shown). The observation that the mutation of *PTC6* in a *ptc1* background is specific and additive suggests that Ptc1 and Ptc6 phosphatases could interact with the TOR pathway at different levels. It has been reported that certain *vps* mutants fail to recover from rapamycin-induced arrest [41]. Since *ptc1* shows diverse phenotypes alike to *vps* mutants [29] we tested the ability to resume growth in rapamycin-treated *ptc1* cells after the drug is removed. We observed that, indeed, the *ptc1* and *ptc1 ptc6* strains do not recover from the drug treatment (Fig. 2B). In contrast, *ptc6* mutants recover nearly as a wild type strain, indicating that, in spite of the common rapamycin-sensitive phenotype, only Ptc1 is essential for resumption of growth after treatment with the drug. This result also supports the notion that Ptc1 and Ptc6 interact with the TOR pathway in a different manner.

It is reasonable to assume that if Ptc1 would be affecting the Ptc6 target(s) in the TOR pathway, its over-expression could rescue the hypersensitivity to rapamycin of the *ptc6* mutant. As shown in Figure 2C, overexpression of native Ptc1 rescues the *ptc1* phenotype and, as previously reported [19], is even able to confer some tolerance to rapamycin, whereas a catalytically impaired version of the phosphatase (D58N) does not. In contrast, overexpression of Ptc1 does not increase at all the tolerance of the *ptc6* mutant to the drug.

**Figure 3:**
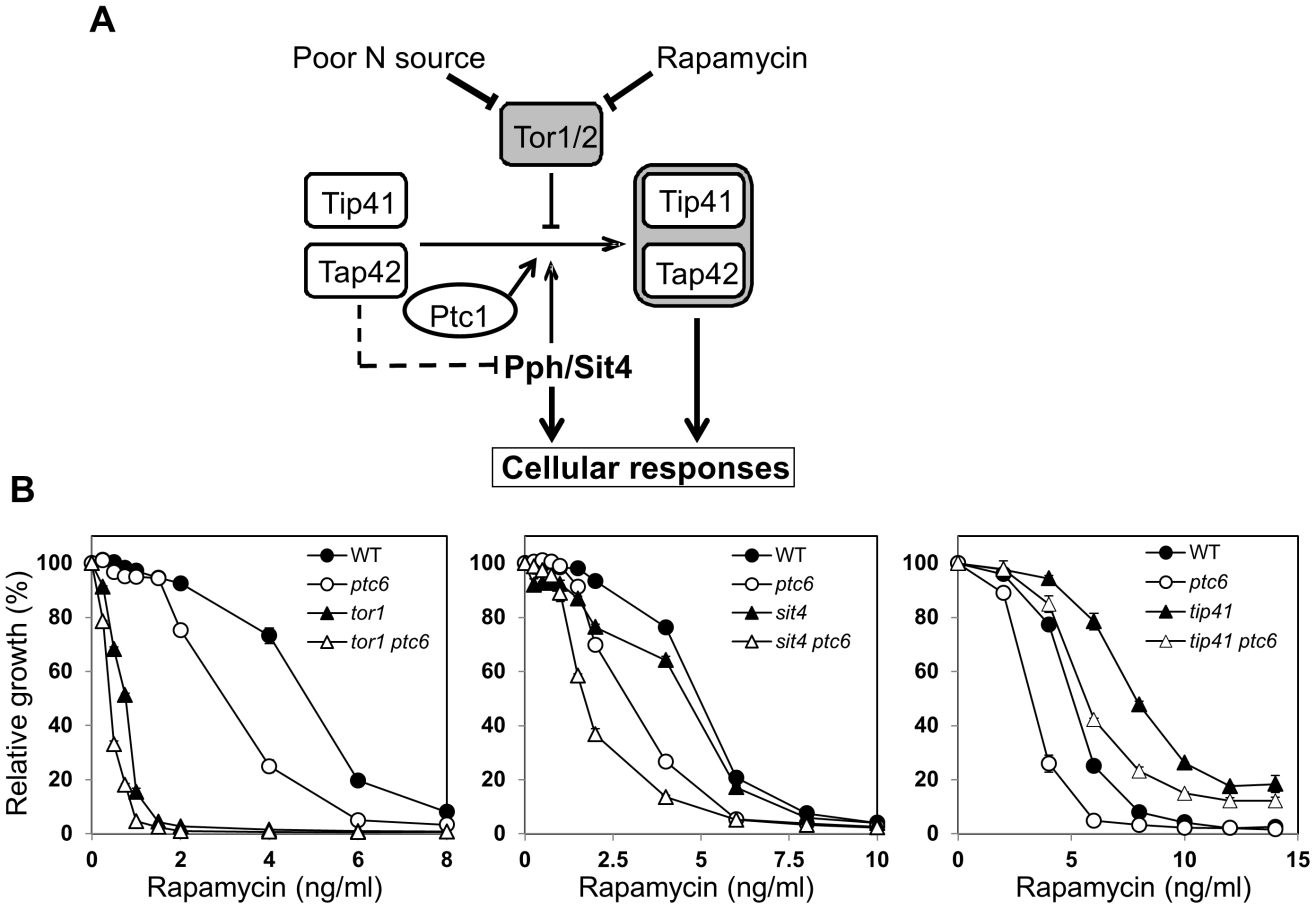
Epistatic analysis of *ptc6* and mutations affecting the TOR pathway. A) A simplified model of signaling through the TOR pathway, focused on the regulation of NCR genes and displaying the proposed role for Ptc1 [19]. B) Growth in the presence of the indicated concentrations of rapamycin of diverse mutants in genes involved in the TOR pathway in combination with deletion of *PTC6*. Relative growth is represented as a percentage respect the growth of each strain in YPD without rapamycin. Six experiments were performed and the mean ± SEM is represented.

Our previous results indicated that Ptc1 acts on the TOR pathway by regulating Tip41 or Sit4 function, since the deletion of *PTC1* in *sit4* or *tip41* cells did not alter the phenotype displayed by these mutants [19]. We now deleted the *PTC6* gene in strains carrying mutations in diverse non-essential components of the pathway (Fig. 3A), and the sensitivity to rapamycin was tested measuring the growth in liquid cultures. As observed in Fig. 3B deletion of *PTC6* increased the rapamycin hypersensitive phenotype of *tor1* cells, similarly to that observed for the deletion of *PTC1* [19]. However, contrary to the previously observed for the *ptc1* mutants, deletion of the *PTC6* gene in the *sit4* or *tip41* backgrounds decreased the tolerance to the drug. Therefore, our data suggest that, in contrast to what happens in the case of *ptc1*, the *sit4* and *tip41* mutations are not epistatic to the *ptc6* mutation.

Therefore, taking together these results indicate that both Ptc6 and Ptc1 are involved in the normal functioning of the TOR signaling pathway but they are affecting different mechanisms.

### Rapamycin-induced mitophagy is blocked in *ptc6* mutants growing on glucose

**Figure 4:**
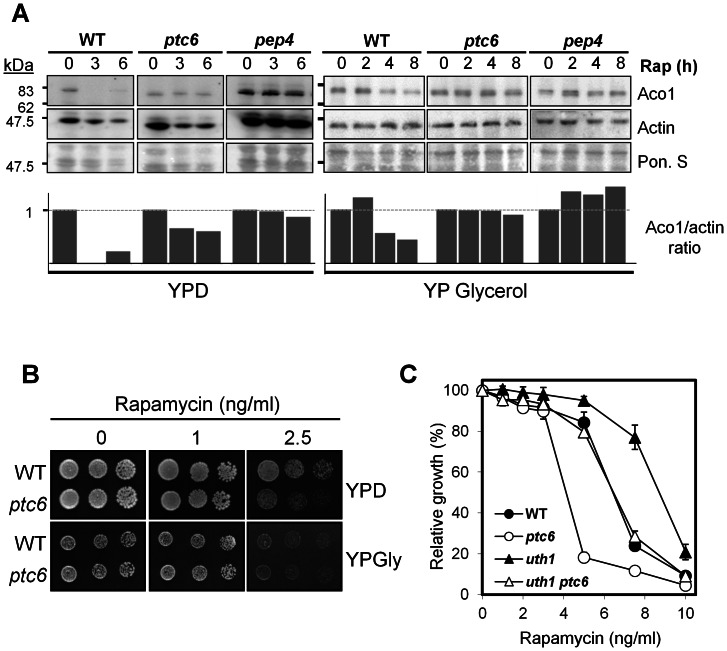
Rapamycin-induced mitophagy is dependent on Ptc6. A) Wild type BY4741 and isogenic *ptc6* and *pep4* derivatives were grown in YPD or YP Glycerol (2%) medium until OD_660_ 1.0 and then rapamycin (200 ng/ml) was added. Samples were taken at the indicated times and 40 µg of total protein from each sample were analyzed by immunoblotting with anti-aconitase and anti-actin antibodies. Ponceau staining (Pon S) is shown as loading control. Quantification of band intensities was performed by the GelAnalyzer software (http://www.gelanalyzer.com) and shown as Aco1/actin signals ratio. B) Wild-type strain BY4741 and the isogenic *ptc6* mutant were spotted onto plates containing glucose (YPD) or glycerol (YPGly) as carbon source, and the indicated concentrations of rapamycin. Growth was monitored after 3 (YPD plates) or 4 days (YPGly plates) of incubation at 28°C. C) The indicated strains were grown in the presence of the different concentrations of rapamycin as described in the legend of Fig. 3B.

One of the events controlled by the TOR signaling pathway, together with Ras-PKA signaling and the general stress response pathways, is mitophagy, a vacuole-dependent mitochondrial degradation process [42]. Several circumstances have been described that lead to yeast mitophagy, including those that inhibit the TOR pathway, such as entry in stationary phase, nitrogen starvation or treatment with rapamycin [11]. By following the degradation of the mitochondrial protein aconitase (Aco1), a well-established method to monitor mitophagy [11], we confirmed that rapamycin treatment in wild type cells exponentially growing in YPD medium induced mitophagy, since the protein was not detectable after 3 h of treatment with the compound (Fig. 4A). In contrast, when *ptc6* mutant cells were exposed to the same concentration of rapamycin, Aco1 was still detectable for at least 6 h, indicating that rapamycin-induced mitophagy in wild type cells is, at least in part, dependent of Ptc6. This behavior is similar to that of cells lacking one of the main vacuolar hydrolases (the proteinase A, encoded by the *PEP4* gene), which have been shown unable to degrade Aco1 when growing on lactate upon rapamycin incubation [43]. Thus, cells lacking Ptc6 have an impaired mitophagy phenotype when treated with rapamycin in medium containing glucose as carbon source. Therefore, our results extend those reported by Tal and coworkers showing that Ptc6 was required for efficient mitophagy in prolonged stationary-phase incubation in medium containing lactate, a non-fermentable carbon source [11]. Ptc6 is also required for the delay in Aco1 degradation observed in wild type cells treated with rapamycin in a non-fermentable carbon source such as glycerol (Fig. 4A). We next asked whether the *ptc6* mutant would be similarly sensitive to rapamycin irrespectively of the carbon source. Interestingly, as shown in Figure 4B, wild type and *ptc6* cells display a similar sensitivity to rapamycin, in spite that the wild type strain still shows some degree of mitophagy and the *ptc6* mutant does not (Fig. 4A), thus indicating that mitophagy and the rapamycin-sensitive phenotype could be dissociated. This notion is further reinforced by the observation that there are strains, such as the *uth1* mutant, that are unable to induce mitophagy in lactate-grown cells challenged with rapamycin, but are tolerant to rapamycin irrespectively of the carbon source ([43], Figure 4C). In fact, deletion of the *PTC6* gene decreases tolerance to rapamycin of the *uht1* mutant (Figure 4C).

### The rapamycin-sensitive phenotype of the *ptc6* mutant is independent of the lack of PDH activity

**Figure 5:**
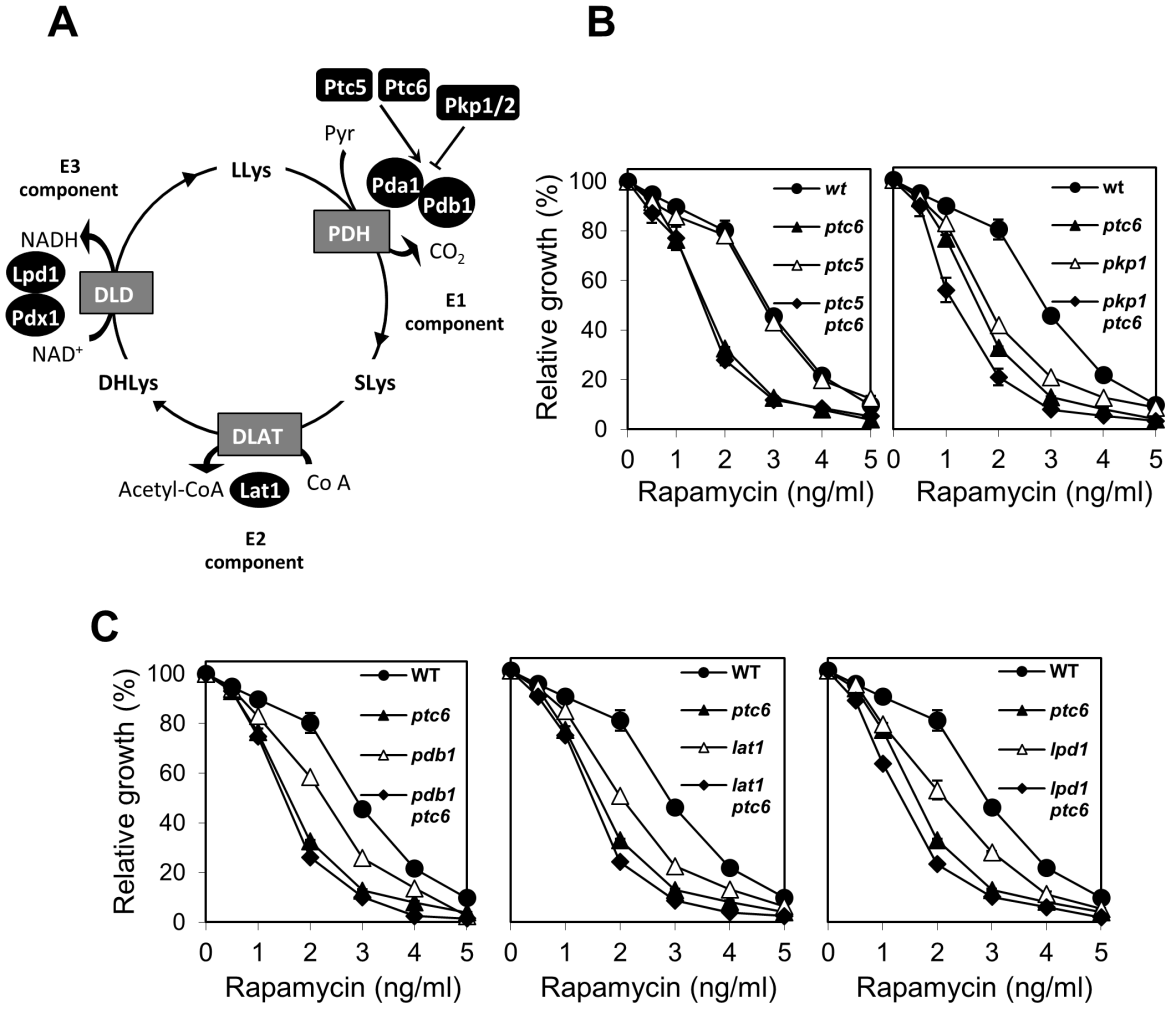
Epistatic analysis of *ptc6* and mutations affecting the PDH complex. A) Schematic representation of the pyruvate dehydrogenase complex based in the information available at the Yeast Biochemical Pathway Database, at the SGD website [51]. The different components of the pyruvate dehydrogenase complex (black ovals) as well as the protein kinases and phosphatases regulating its activity (black rectangles) by targeting Pda1, a component of the E1 complex, are shown. Grey rectangles denote the following enzymatic activities: Pyruvate dehydrogenase (PDH), Dihydrolipoyl lysine acetyltransferase (DLAT) and Dihydrolipoyl dehydrogenase (DLD). The following abbreviations have been used to label the reaction products: Pyr, Pyruvate, LLys (N^6^-(lipoyl)lysine), SLys (N^6^-(S-acetyldihydrolipoyl)lysine) and DHLys (N^6^-(dihydrolipoyl)lysine). B) Growth in the presence of the indicated concentrations of rapamycin of diverse strains lacking genes involved in regulating the E1 component of the pyruvate dehydrogenase complex in combination with the deletion of *PTC6*. Growth is represented as a percentage respect the growth of the each strain in YPD without rapamycin. Data are mean ± SEM from six experiments. C) Rapamycin sensitivity of diverse mutants lacking structural components of the PDH complex and the *PTC6* gene. Experimental conditions are identical to those described above.

The only cellular target for Ptc6 described so far is Pda1, which is also dephosphorylated by Ptc5. As shown in Figure 5A, five proteins form the three structural components of the PDH complex. In addition, two protein phosphatases (Ptc6 and Ptc5) and two protein kinases (Pkp1 and Pkp2) are regulatory elements, responsible for the increase and decrease of PDH activity, respectively. Interestingly, Gey and coworkers demonstrated that lack of Ptc6 causes a dramatic drop in the PDH activity and it has been reported that deletion Pda1 [13] or Pdb1 [18] results in sensitivity to rapamycin. Therefore, it could be speculated that a link between the rapamycin-sensitive phenotype of *ptc6* cells and the role of the phosphatase in activating PDH activity could exist. To test this possibility we evaluated the sensitivity to rapamycin of mutants in genes involved in regulation of PDH activity in the presence and in the absence of the *PTC6* gene. As shown in Figure 5B, deletion of *PTC6* in the *ptc5* background (which is not rapamycin-sensitive) decreases tolerance to the drug to achieve *ptc6* levels. Remarkably, we observe that the *pkp1* strain, which does not show a decrease in PDH activity [13], [18], is also hypersensitive to rapamycin and that deletion of *PTC6* further decreases the tolerance to the drug. Similarly, lack of Ptc6 decreases tolerance to rapamycin in cells lacking structural components of the PDH complex, such as in the *pdb1*, *lat1* or *lpd1* strains (Figure 5C). Therefore, deletion of *PTC6* decreases rapamycin tolerance irrespectively of an increase or decrease of PDH activity, thus suggesting that decreased PDH activity is not the cause of the hypersensitivity to rapamycin described for the *ptc6* mutant. Because the slightly increased sensitivity to rapamycin observed in mutant cells lacking either structural components of the PDH complex or the *PKP1* gene, we cannot discard a role of these components in the pathway regulated by rapamycin.

### The rapamycin-induced transcriptional response is attenuated in *ptc6* mutant cells

**Figure 6:**
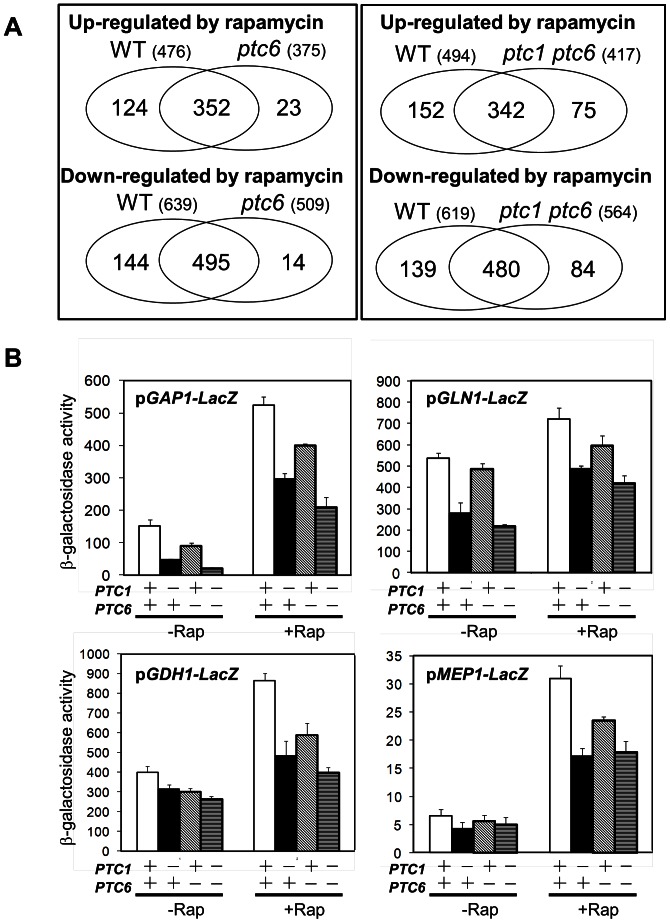
Cells lacking Ptc6 have an attenuated transcriptional response to rapamycin. A) Venn diagram showing the number of genes whose expression was considered to be induced or repressed by rapamycin in wild type and *ptc6* cells (left panel) or wild type and *ptc1 ptc6* cells (right panel). Datasets with valid data in each case contained 3332 and 3810 genes, respectively. B) Decreased response of diverse NCR-sensitive genes to rapamycin treatment. The indicated constructs were introduced into wild type BY4741 and the indicated derivatives, and cells were treated with 200 ng/ml rapamycin (Rap) for 60 (for *GAP1*, *GLN1* and *GDH1* promoters) or 90 min (for *MEP1*). Control cells (- Rap) received only the solvent. β-Galactosidase activity was measured as indicated in the text. Data are mean ± SEM from six independent clones.

Short-term treatment of wild type cells with rapamycin results in strong remodeling of gene expression. To identify the effect of *ptc6* mutation in the global expression pattern after rapamycin treatment, we performed microarray experiments comparing the rapamycin-induced transcriptional response of wild type and *ptc6* mutants. Considering only the genes with valid data in both experiments (3332 genes) we found that rapamycin caused changes in the expression levels of 1115 genes (33.4%) in wild type cells (476 up-regulated and 639 down-regulated). In *ptc6* mutant cells, rapamycin changed the levels of 884 transcripts (26.5%), being the number of up-regulated and down-regulated genes of 375 and 509, respectively (Fig. 6A, upper panel). In the case of the *ptc1 ptc6* mutant, from the total number of genes with valid data (3355), the rapamycin-induced genes were 417 (12.4%) and the down-regulated ones were 564 (16.8%) (Fig. 6A, lower panel). These figures suggest that lack of Ptc6 may cause an attenuation of the transcriptional response to rapamycin. The alterations provoked by the *ptc1* and *ptc6* mutations in the response induced by rapamycin were verified in a set of four rapamycin-responsive, nitrogen catabolite repression (NCR) regulated genes (*GAP1*, *GLN1*, *GDH1* and *MEP1*), required for adaptation to non-preferred nitrogen sources and whose expression is controlled by the Gln3 transcription factor. As shown in Fig. 6B, when cells were challenged with rapamycin, it became evident that the response of these genes to the drug was attenuated in Ptc6-deficient cells, although somewhat less than in *ptc1* cells. The transcription attenuation of the *GAP1* and *MEP1* genes in response to rapamycin in the *ptc6* strain was also validated by RT-PCR (not shown).

To explore the extent of the transcriptional attenuation in the response to rapamycin caused by the *ptc6* mutation, we plotted the transcriptional changes (log_2_ values) for wild type (X axis) and *ptc6* (Y axis) after rapamycin treatment, and the value of the slope of the fitted line obtained by simple linear regression was taken as an “attenuation index” (Figure S1). Therefore, if the global responses to the drug in both strains were similar, the expected slope value would be close to unity, whereas a weakened response in the *ptc6* cells would decrease this index. The values of the slope for the genes consider up- ad down-regulated by rapamycin in *ptc6* cells respect wild type cells were 0.8186 and 0.7424. As a reference, the corresponding indexes for the same treatment with rapamycin in *ptc1* respect wild type cells were 0.7502 and 0.9268, respectively [19]. We also performed a similar calculation with the *ptc1 ptc6* cells, and the corresponding slopes were 0.6671 and 0.8869 (Figure S1). Thus, changes in the transcriptional profiles in response to rapamycin were attenuated in all three mutants analyzed, being this attenuation more relevant for the down-regulated genes in the *ptc6* strain, and for the up-regulated genes in the *ptc1 ptc6* strain.

One of the cellular responses to rapamycin is the translocation to the nucleus of the Msn2 transcription factor that collaborated in the regulation of the general stress response. We have already described that lack of Ptc1 prevents the nuclear translocation of Msn2 in response to rapamycin [19]. When similar experiments were performed with *ptc6* mutant cells, we observed only a moderate decrease in the number of cells with nuclear localization of Msn2, when compared to wild type cells (Figure S2A). Consistently, when the attenuation in the expression change in *ptc6* cells of genes known to be under the regulation of Msn2/Msn4 was compared with that of genes independent of these transcriptional factors, no significant differences were observed (Figure S2B). Cells lacking both genes, *PTC1* and *PTC6*, however, resulted in a stronger prevention of the nuclear localization that the observed for *ptc1* cells.

### Ptc6 is important for the rapamycin-induced down-regulation of the genes involved in ribosome biogenesis

We next investigated if the *ptc6* mutation affected specific gene families whose expression was altered by rapamycin-treatment. We have found that the expression level of 476 genes was up-regulated by rapamycin in wild type cells, when genes with valid data for the *ptc6* strain were considered. The most relevant category among these genes was the metabolism of aminoacids (67 genes, p-value: 7.65E-22). Only 86 genes were, at some extent, dependent of Ptc6. The additional 390 genes were up-regulated in a Ptc6-independent manner. Among them, the family of genes involved in the metabolism of aminoacids was the most relevant (55 genes, p-value: 7.43E-18).

Concerning the 639 genes whose expression was found down-regulated by rapamycin in wild type cells we observed, in agreement with previous reports, a very strong predominance of genes related to protein synthesis (217 genes, p-value 4.98E-102), particularly those involved in ribosomal biogenesis and translation (Table S4).

We also observed a significant enrichment in genes related to transcription (144 genes, p-value 3.09E-5), predominantly those associated to rRNA processing (70 genes, p-value 3.87E-22). When the level of dependence on Ptc6 for transcriptional down-regulation was examined, we found 290 genes (45.4%) exhibiting some degree of dependence (9 totally, 83 strong and 198 weakly dependent on Ptc6). Interestingly, while globally considered, genes related to protein synthesis did not exhibit a particular trend regarding Ptc6-dependence, the down-regulation of the subset corresponding to ribosome biogenesis showed a tendency to be Ptc6-dependent, in particular those coding for ribosomal proteins (p-value of 4.68E-41 for the Ptc6-dependent *vs* 6.14E-24 for the Ptc6-independent). In general, the degree of dependence for this gene family was weak (Table S4). In contrast, rapamycin-repressed genes related to translation turned out to be largely independent of Ptc6. When genes involved in transcription were considered, we observed a predominance of Ptc6-dependent genes (Table S4). Remarkably, this tendency is not general for all functional subcategories, but it is particularly strong for genes related to rRNA processing (53 genes, p-value 5.34E-27, *vs* 17 genes, p-value 4.92E-2, for independent genes). Attenuation of repression of genes within this category in response to rapamycin was, in many cases, largely dependent on the presence of Ptc6, with predominance of TD- or SD- dependent genes (Table S4).

### Ptc6 is not required for rapamycin-induced dephosphorylation of Sch9

**Figure 7:**
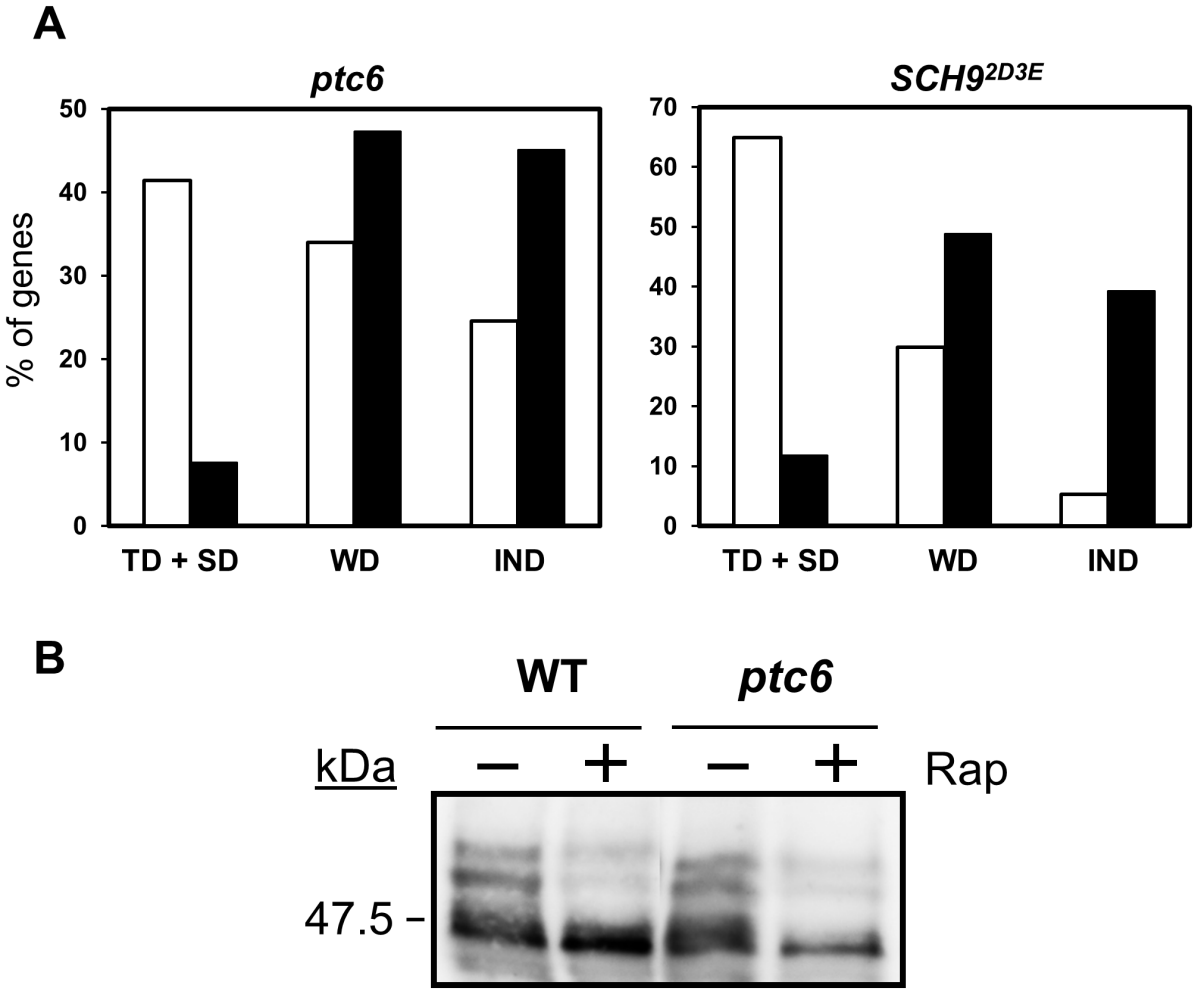
Ptc6 does not influence Sch9 phosphorylation in response to rapamycin. A) Representation of the percentages of the genes down-regulated by rapamycin involved in rRNA processing (open bars) or ribosomal proteins (closed bars) whose repression was totally dependent/strongly dependent (TD/SD), weakly dependent (WD) or independent (IND) on the presence of Ptc6 (left panel, data from this work). The same gene families were also classified according to the level of dependence on Sch9 in response to rapamycin (right panel). Data for Sch9 was taken from [22] and processed according to the same method used for establishing Ptc6 dependence. B) Exponentially growing wild type and *ptc6* mutant cells expressing HA-tagged Sch9 were treated with rapamycin (200 ng/ml) for 30 min, as detailed in the Materials and Methods section. TORC1 phosphorylation of the C-terminal portion of Sch9-HA, that leads to activation of Sch9, was determined in the lysates by NTCB treatment and immunodetected with anti-HA antibodies.

It is known that TORC1 directly phosphorylates Sch9, a member of the AGC family of protein kinases, thus triggering expression of genes involved in ribosome biogenesis [22]. Interestingly, comparison of the response profile to rapamycin of cells carrying a constitutively active version of Sch9, that mimics a TORC1-phosphorylated form (*SCH9^2D3E^* allele) with that of the native protein reveals an attenuation of the response to drug that is qualitatively and quantitatively similar to that observed here for *ptc6* mutants. Thus, when the “attenuation index” described above was calculated for all genes repressed after 90 min of exposure to rapamycin, a value of 0.706 was obtained, in close agreement with that obtained for *ptc6* cells (0.742). The similarity extends to the differential level of dependence when gene families were considered. Repression of genes coding for proteins involved in rRNA processing showed an important grade of dependence for both Ptc6 and Sch9. These dependences were weaker in both cases for the set of genes related to the ribosomal protein synthesis (Figure 7A). Down-regulation of the genes of the regulon Ribi was also found attenuated in both mutants (Figure S3).

This raised the possibility that Ptc6 might exert its function in the TOR pathway by directly or indirectly dephosphorylating and inactivating Sch9. To test this, we examined the phosphorylation state of Sch9 in wild type cells and *ptc6* mutants in response to rapamycin (Figure 7B). However, our results indicate that the phosphorylation state of Sch9 remains unchanged irrespective of the presence or absence of Ptc6, suggesting that the phosphatase is not responsible for the control of the kinase.

### Down-regulation of the genes controlled by Ifh1 in response to rapamycin requires Ptc6

**Figure 8:**
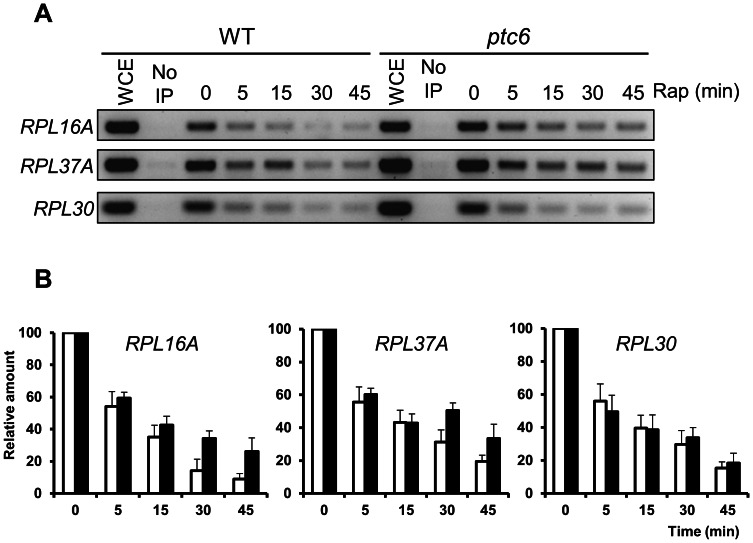
Lack of Ptc6 influences Ifh1 release from Fhl1-regulated promoters. A) Chromatin immunoprecipitation experiments were performed using W303-1A wild type (WT) and *ptc6* mutant strains carrying a MYC-tagged *IFH1* version. Tagging of Ifh1 did not affect the sensitivity to rapamycin of *ptc6* cells (not shown). After the addition of rapamycin (200 ng/ml) samples were taken at the indicated times. The immunoprecipitated DNA was used as a template for PCR by using specific primers for the promoters of the indicated genes (see Table S1). The PCR products were resolved in 2.5% agarose gel. Representative results from one out of three independent experiments are shown. WCE, Whole cell extract. B) Quantification of the relative amount of the PCR-products obtained, for the indicated promoters of the ribosomal protein-encoding genes shown above, for wild type cells (open bars) and *ptc6* mutant cells (closed bars). Four independent experiments were performed. Error bars represent the standard deviation.

According to our microarray data, *ptc6* mutant cells showed a weakened down-regulation of the gene expression caused by the inhibition of TOR for genes encoding cytosolic RP (ribosomal proteins). These results were also validated by quantitative RT-PCR (Figure S4) and semiquantitative RT-PCR (not shown) for three RP and a member of the Ribi regulon. Therefore, we hypothesized that Ptc6 might be involved in the regulation of expression of these genes. Ifh1 is a co-activator of the Forkhead-like Fhl1 transcription factor that is recruited to the promoters of the ribosomal protein encoding genes during optimal growth conditions by Fhl1 and is absent when transcription is repressed [44]. Rapamycin treatment is one of the known situations that decrease the binding of Ifh1 to RP promoters [45]. Therefore, it was conceivable that Ptc6 might influence binding of Ifh1 to its target promoters. To test this hypothesis we examined the binding of a myc-tagged version of Ifh1 to the promoters of diverse RP genes after rapamycin treatment in both wild type and *ptc6* cells by chromatin-immunoprecipitation (ChIP) followed by PCR assays. For all three RP genes analyzed, we detected a marked decrease (50–60%) of Ifh1 binding to the promoters after 5 min of rapamycin treatment in wild type cells and this effect was even more prominent at longer times (∼20% of binding after 45 min). In *ptc6* cells, however, Ifh1 binding was clearly higher for the promoters of *RPL16A* and *RPL37A*, particularly after 30 minutes of exposition to rapamycin (Figure 8). It is worth noting that *RPL16A* was found to be strongly dependent on Ptc6 according to our microarray data, whereas for *RPL30* the effect of lack of the phosphatase was only marginal. Although no microarray data for *RPL37A* was available, quantitative RT-PCR results (Figure S4) indicate a significant effect of the *ptc6* mutation on *RPL37A* mRNA accumulation. This suggests that the attenuation of the repression of the genes encoding RP described for the *ptc6* mutant cells could be due, at least in part, to a defect in releasing Ifh1 from their promoters.

## Discussion

We have accomplished an extensive characterization of the phenotypes shown by Ptc6-deficient cells, identifying novel phenotypes for the *ptc6* mutant cells not reported in a previous work that characterized mutants in non-essential catalytic subunits of protein phosphatases [46]. The transcriptional profiling of the *ptc6* mutants is more similar to that of the *ptc1* strain than to other *ptc*-deficient cells, such as *ptc5* (Figure 1C). This is somewhat surprising, since it has been proposed that *ptc5* and *ptc6* share the same subcellular location (mitochondria) and a regulatory function on the PDH complex [13]. In spite of the similarity of their transcriptional profiles, most *ptc6* phenotypes differ from those displayed by *ptc1* mutant. For example, *ptc1* cells were hypersensitive to alkaline pH as well as to high calcium concentrations [29], phenotypes that are landmarks of deficient vacuolar function [47]. Indeed, cells lacking *PTC1* displayed highly fragmented vacuoles. In contract, *ptc6* cells were not sensitive to either high pH or calcium and, accordingly, their vacuolar morphology was normal. Another relevant difference between *ptc6* and *ptc1* strains is that *ptc6* cells are tolerant to both, cell-wall stressors and excess of LiCl, while *ptc1* cells are sensitive. However, *ptc1* and *ptc6* cells do share some phenotypes, such as hypersensitivity to rapamycin, suggesting that Ptc6 may be involved in the TOR pathway. Nevertheless, several lines of evidence suggest that both enzymes impact the TOR pathway at different levels: i) the rapamycin-sensitive phenotypes of the *ptc1* and *ptc6* mutations are additive. ii) overexpression of *PTC1* cannot rescue the rapamycin-sensitive phenotype of the *ptc6* strain, iii) exposure to high concentrations of rapamycin causes an irreversible halt in growth in *ptc1* mutants, whereas Ptc6-deficiente cells can survive when the drug is removed from the medium, and iv) the *ptc6* mutation does not shown the epistatic relationships with relevant components of the TOR pathway displayed by the *ptc1* mutation [19].

Early work on Ptc6 [13] suggested that the rapamycin-sensitive phenotype of the *ptc6* mutant could derive from the proposed role of the phosphatase in activating PDH activity. However, we cannot find a relationship between the effect of a given mutation on PDH activity and the effect of such mutation on rapamycin sensitivity. For instance, using liquid cultures we observe that lack of *ptc6*, which should inhibit Pda1, causes a stronger rapamycin-sensitive phenotype than deletion of the *PDA1* gene (not shown). Similarly, deletion of both *PTC5* on the *ptc6* background, which should fully eliminate the ability to dephosphorylate the PDH complex, does not result in increased sensitivity to rapamycin. More importantly, lack of *PKP1*, encoding a Pda1 kinase, also results in sensitivity to rapamycin which is further aggravated by lack of Ptc6 (Figure 5B). Therefore, our results do not support the hypothesis linking the sensitivity to rapamycin of the *ptc6* mutant and the role of this phosphatase in the regulation of PDH activity. Similarly, our data also demonstrate that whereas the rapamycin-sensitive phenotype of the *ptc6* mutant is dependent on the carbon source, it cannot be linked to the occurrence of mitophagy upon exposure to rapamycin (Figure 4).

We observed that the expression of genes coding for proteins involved in both the ribosome biogenesis and the rRNA processing were down-regulated in a Ptc6 dependent manner (Table S4). It is known that repression of these sets of genes after inhibition of the TOR pathway may be under the control of Sch9 [22], [48] and that Sch9 is activated by phosphorylation [22]. Therefore, it could be hypothesized that Ptc6 might directly or indirectly promote Sch9 dephosphorylation and deactivation. However, we did not detect changes in the phosphorylation state of Sch9 in the absence of Ptc6 and, consequently, we conclude that Ptc6 must have targets other than Sch9. This would be in agreement with the fact that, whereas it has been reported that Sch9 mediates TORC1 regulation of transcription initiation [22], we find that rapamycin-induced repression of most genes related to this function is largely independent of the presence of Ptc6 (Table S4). Similarly, whereas Sch9 is not involved in the expression of Gln3-regulated genes [22], we observe attenuated expression of this kind of genes in *ptc6* cells (Figure 6B), indicating the existence of alternative Ptc6 cellular targets.

Expression of ribosomal protein- and pre-rRNA processing-encoding genes is also under the control of the Fhl1 forkhead transcription factor [44], [49]. When the TOR pathway is active, the coactivator Ifh1 binds to Fhl1, thus promoting expression of Fhl1-regulated genes. Inactivation of the TOR pathway results in Yak1-mediated phosphorylation of the Crf1 corepressor, promoting its binding to Fhl1, displacement of Ifh1, and switching off transcription of the Fhl1-regulated genes [49]. We observe that in cells lacking Ptc6, rapamycin-induced release of Ifh1 from Fhl1-regulated promoters is delayed or abolished. Since failure to effectively release Ifh1 from its target promoters would interfere with transcriptional switch off, this might contribute to explain, at least in part, the attenuation of rapamycin-induced repression of genes involved in ribosome biogenesis. The mechanisms for this effect would be open to conjecture. One possibility is that Ptc6 may regulate the phosphorylation state of Crf1. If so, Ptc6 could not act as a Crf1 phosphatase, since lack of Ptc6 would lead to hyperphosphorylation of Crf1 and this would lead to the potentiating of the repressor effects of rapamycin on target genes expression. It would be possible, however, that Ptc6 could negatively regulate the input of the TOR pathway on Yak1 activation. It must be noted that in vivo phosphorylation of Ifh1 has been reported in high-throughput studies. Interestingly, it has been recently shown that Ifh1 can be phosphorylated in vitro by Yak1 [50]. Therefore, the hypothetic regulation of Yak1 activity by Ptc6 could also impact on Ihf1 itself. In addition, it has been shown that Ifh1 is in a complex with casein kinase 2 (CK2), Utp22 and Rrp7 (CURI complex) are implicated in the processing of pre-rRNA and that CK2 phosphorylates in vitro Ifh1 [44]. Therefore, CK2-mediated phosphorylation of Ifh1 could also be a target for Ptc6 function.

## Supporting Information

Figure S1 **Linear regression analyses were used to estimate the transcriptional attenuation caused by the lack of** ***ptc6*** **or** ***ptc1*** **and** ***ptc6*** **.** Linear regression analysis of the plotted values for the changes in the level of expression triggered by rapamycin in wild type and in the indicated mutant strains. The obtained equation is indicated for each case. A) Set of 476 genes up-regulated by rapamycin in wild type cells plotted against their expression value in the *ptc6* mutant. B) Set of 639 genes down-regulated by rapamycin in wild type cells plotted against their expression value in the *ptc6* mutant. C) Set of 494 genes up-regulated by rapamycin in wild type cells plotted against their expression value in the *ptc1 ptc6* mutant. D) Set of 619 genes down-regulated by rapamycin in wild type cells plotted against their expression value in the *ptc1 ptc6* mutant.
(TIF)

Figure S2 **Lack of Ptc6 moderately affects rapamycin-induced down-regulation of the Msn2/Msn4-controled genes.** A) Intracellular localization of Msn2-GFP at the indicated times after addition of rapamycin to the cultures of WT, *ptc1*, *ptc6* and *ptc1 ptc6* cells. Cells from each strain were distributed into three categories according to the intracellular localization of Msn2-GFP: cytosolic (black bars), cytosolic and nuclear (grey bars) and nuclear (white bars). B) Plots of the log_2_ values for the changes in the level of expression consequence of the treatment with rapamycin in both WT (dots) and *ptc6* strains (closed triangles) for the 150 most up-regulated genes (top panel) and 150 most down-regulated (bottom panel) genes in the WT strain. The expression values for the genes documented targets of Msn2 or Msn4 described in YEASTRACT [53] plus those identified elsewhere [54] in *ptc6* cells are denoted as open squares.
(TIF)

Figure S3 **Comparison of transcriptional changes in** ***ptc6*** **mutants and cells expressing a constitutive active version of Sch9**. Representation of the averages of the changes in the level of expression (in log_2_) induced by rapamycin for the genes included in the specified categories that constitute the Ribi regulon [55]. Left panel shows data corresponding to yeast (W303 background) expressing normal and constitutively active Sch9 (*SCH9* ^2D3E^) after 90 min treatment with rapamycin obtained from the GEO database (series reference GSE7660) [22]. Right panel shows data from the experiments described in this work.
(TIF)

Figure S4 ***ptc6*** **mutant cells exhibit an attenuation in the repression of genes involved in the ribosome biogenesis caused by rapamycin.** Wild type BY4741 and its isogenic *ptc6* derivatives were grown in YPD and treated with rapamycin as described in Figure 4A. Samples were collected at different times and total RNA was prepared. Quantitative RT-PCR were performed by duplicate using specific oligonucleotides, as described in the Materials and Methods section, and the levels of expression of the RNA for the specified genes in wild type BY4741 (empty bars) and *ptc6* mutant cells (filled bars), after actin normalization, are represented as a percentage respect the quantity of RNA in untreated cells. Error bars represent the standard deviation.
(TIF)

Table S1 **Oligonucleotides used in this study.**
(DOCX)

Table S2 **Genes up-regulated in** ***ptc1 ptc6*** **cells.**
(DOCX)

Table S3 **Genes down-regulated in** ***ptc1 ptc6*** **cells.**
(DOCX)

Table S4 **Major functional categories of genes down-regulated by rapamycin.** The set of genes in each category (according to the MIPS Functional Catalogue Database) is classified as affected (dependent) or unaffected (independent) by the absence of Ptc6. Ptc6-dependent genes are further classified into totally plus strongly dependent (TD+SD) and weakly dependent (WD).
(XLS)
