# Supplementary figures and images for: Ptc6 Is Required for Proper Rapamycin-Induced Down-Regulation of the Genes Coding for Ribosomal and rRNA Processing Proteins in S. cerevisiae
Source: PLoS One. 2013 May 21;8(5):e64470. doi: 10.1371/journal.pone.0064470 (PMC3660562; doi:10.1371/journal.pone.0064470)

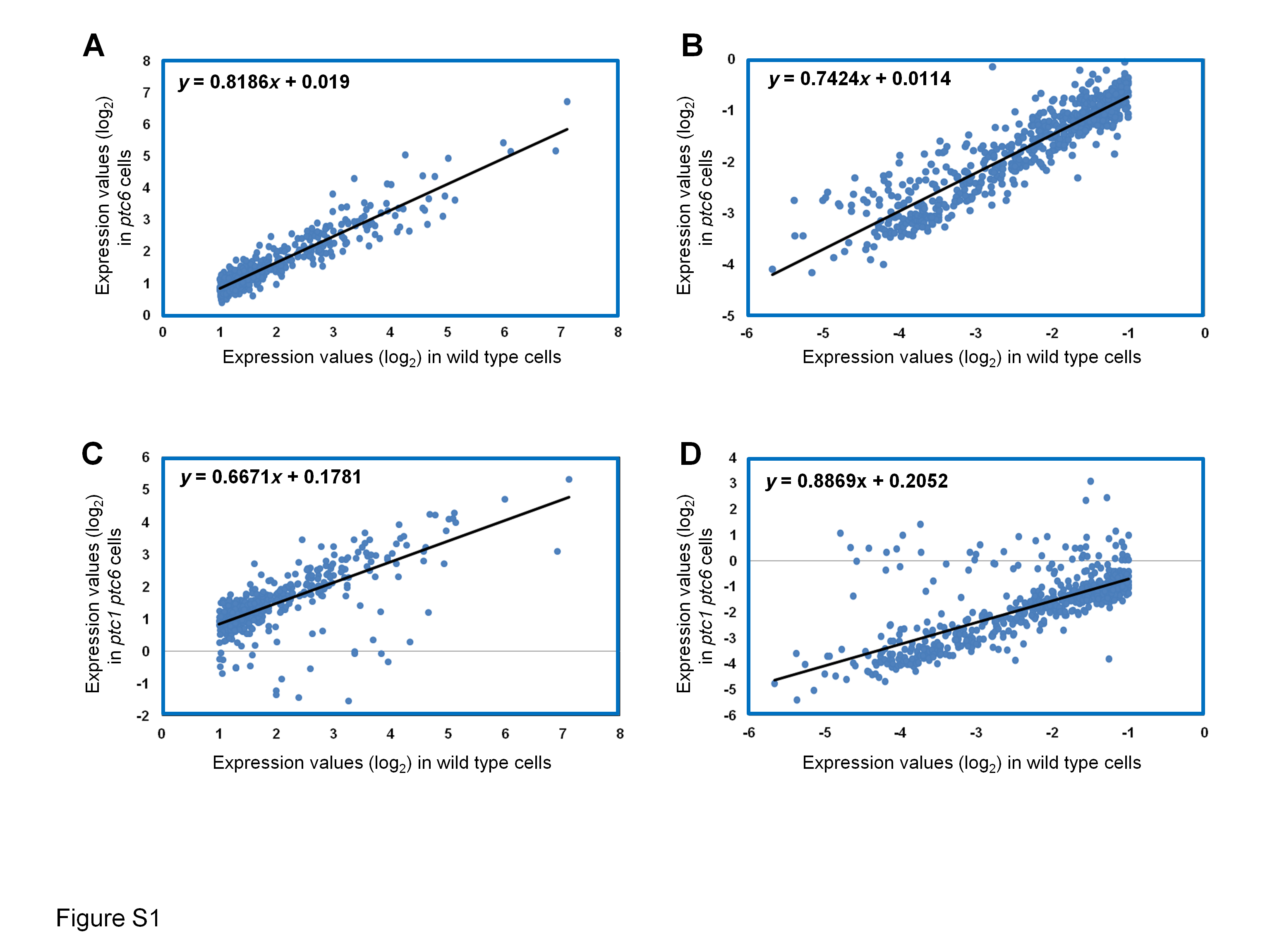

Supplement: Figure S1 — Linear regression analyses were used to estimate the transcriptional attenuation caused by the lack of ptc6 or ptc1 and ptc6 . Linear regression analysis of the plotted values for the changes in the level of expression triggered by rapamycin in wild type and in the indicated mutant strains. The obtained equation is indicated for each case. A) Set of 476 genes up-regulated by rapamycin in wild type cells plotted against their expression value in the ptc6 mutant. B) Set of 639 genes down-regulated by rapamycin in wild type cells plotted against their expression value in the ptc6 mutant. C) Set of 494 genes up-regulated by rapamycin in wild type cells plotted against their expression value in the ptc1 ptc6 mutant. D) Set of 619 genes down-regulated by rapamycin in wild type cells plotted against their expression value in the ptc1 ptc6 mutant. (TIF) [file pone.0064470.s001.tif]
