# Supplementary material for: Ptc6 Is Required for Proper Rapamycin-Induced Down-Regulation of the Genes Coding for Ribosomal and rRNA Processing Proteins in S. cerevisiae
Source: PLoS One. 2013 May 21;8(5):e64470. doi: 10.1371/journal.pone.0064470 (PMC3660562; doi:10.1371/journal.pone.0064470)
Supplement: Table S1 — Oligonucleotides used in this study. (DOCX) [file pone.0064470.s005.docx]

**Table S1. Oligonucleotides used in this study**.

| **Name** | **Sequence (from 5’ to 3’)** | **Comments** |
| --- | --- | --- |
| 5'-PTC6-disr_nat | CTGCAATCGGGGCAATTAAGCATCAGAAGAGGGGAATTTGATGCGCTGCAGGTCGACGGATC | 5’ for *ptc6::nat1* cassette amplification from pAG25 |
| 3'-PTC6-disr_nat | CTTGTTTCCACCCAGGGGGGTGTTTAGTTTAATTTTGTTTCGGCATAGGCCACTAGTGGATC | 3’ for *ptc6::nat1* cassette amplification from pAG25 |
| 5’-PTC6 | CCTGTGGATGACCCATGGCG | 5' to verify *ptc6::nat1* disruption (-720 from ATG) |
| nat1-3' | GTGAAGGACCCATCCAGTGC | 3’ to verify *ptc6::nat1* disruption (+92 from ATG of *nat1*) |
| 5’-GAP1_RT | GGTTACTAGCCTTGTCTGGG | 5' RT-PCR for *GAP1* (+1352 from ATG) |
| 3’-GAP1_RT | CGGGGAATACAGCAACGTAG | 3' RT-PCR for *GAP1* (+1561 from ATG) |
| 5’-MEP1_RT | GCAGATCGCTTACATTGCCG | 5' RT-PCR for *MEP1* (+1116 from ATG) |
| 3’-MEP1_RT | CAGAGCGTTGTGAATCTTCG | 3’ RT-PCR for *MEP1* (+1317 from ATG) |
| RT-ACT1 up2 | TGCTGTCTTCCCATCTATCG | 5’ RT-PCR and q-RT-PCR for *ACT1* (+84 from ATG) |
| RT-ACT1 do2 | ATTGAGCTTCATCACCAAC | 3’ RT-PCR and q-RT-PCR for *ACT1* (+178 from ATG) |
| RPL37A_F | CCCGTTCTCTAGGCTGATGG | 5’ ChIP for *RPL37A* (-225 from ATG) |
| RPL37A_R | GACAGACCGACGGACCACAC | 3’ ChIP for *RPL37A* (-159 from ATG) |
| RPL30_F | CCCGTCTATTCTCGTGTCGT | 5’ ChIP for *RPL30* (-499 from ATG) |
| RPL30_R | TGCTAGATGGGGGTTCAGTC | 3’ ChIP for *RPL30* (-448 from ATG) |
| RPL16A_F | GATTATTTTCATGATACTTCG | 5’ ChIP for *RPL16A* (-353 from ATG) |
| RPL16A_R | CAGGATTTGTTCTGCATTGG | 3’ChIP for *RPL16A* (-290 from ATG) |
| 5’-RPL37A_RT | CCTGTGGTTATCCAGCTGC | 5' q-RT-PCR for *RPL37A* (+466 from ATG) |
| 3'-RPL37A_RT | GCCTTAGAAGCAGAGCCGG | 3' q-RT-PCR for *RPL37A* (+616 from ATG) |
| 5’-RPL30_RT | CCGCTAACACTCCAGTTTTG | 5' q-RT-PCR for *RPL30* (+364 from ATG) |
| 3'-RPL30_RT | CTCTGAATAACTTACCGACAGC | 3' q-RT-PCR for *RPL30* (+489 from ATG) |
| 5’-RPL16A_RT | GAGAGTTGTTGTCCCACAAGCA | 5' q-RT-PCR for *RPL16A* (+638 from ATG) |
| 3'-RPL16A_RT | TAGCATTAGCAGAGGCAACTTTC | 3' q-RT-PCR for *RPL16A* (+840 from ATG) |
| 5'-RPF2_RT | CATGGGTGATAAATTGGGTAG | 5' q-RT-PCR for *RPF2* (+801 from ATG) |
| 3'-RPF2_RT | CTTCATCTACCTCTCCATCAC | 3' RT-PCR for *RPF2* (+931 from ATG) |
