# Supplementary material for: Ptc6 Is Required for Proper Rapamycin-Induced Down-Regulation of the Genes Coding for Ribosomal and rRNA Processing Proteins in S. cerevisiae
Source: PLoS One. 2013 May 21;8(5):e64470. doi: 10.1371/journal.pone.0064470 (PMC3660562; doi:10.1371/journal.pone.0064470)
Supplement: Table S2 — Genes up-regulated in ptc1 ptc6 cells. (DOCX) [file pone.0064470.s006.docx]

**Table S2. Genes up-regulated in *ptc1 ptc6* cells.**

| **GENE** | **-Fold increase** |  |  | **GENE** | **-Fold increase** |
| --- | --- | --- | --- | --- | --- |
| *HSP26* | **15.32** |  |  | *FLC2* | **2.38** |
| *HSP12* | **12.36** |  |  | *GLK1* | **2.35** |
| *KDX1* | **10.11** |  |  | *BDH2* | **2.35** |
| *AMS1* | **7.39** |  |  | *HXK1* | **2.31** |
| *FMP33* | **7.30** |  |  | *GAD1* | **2.31** |
| *NQM1* | **6.82** |  |  | *GOR1* | **2.31** |
| *HXT5* | **5.53** |  |  | *YPL088w* | **2.30** |
| *GPG1* | **5.14** |  |  | *ARN1* | **2.30** |
| *FIT3* | **5.08** |  |  | *YMR090w* | **2.28** |
| *SRL3* | **4.84** |  |  | *YBR241c* | **2.27** |
| *CRG1* | **4.81** |  |  | *SPI1* | **2.26** |
| *YMR103c* | **3.97** |  |  | *SSE2* | **2.23** |
| *YOR289w* | **3.66** |  |  | *SED1* | **2.22** |
| *YFL015c* | **3.64** |  |  | *MEP2* | **2.19** |
| *PRM5* | **3.57** |  |  | *PFK26* | **2.16** |
| *RTC3* | **3.49** |  |  | *UGA2* | **2.16** |
| *PGM2* | **3.44** |  |  | *FBP26* | **2.14** |
| *AFR1* | **3.37** |  |  | *YCL049c* | **2.14** |
| *PST1* | **3.36** |  |  | *AGP2* | **2.12** |
| *SOL4* | **3.28** |  |  | *YLR194c* | **2.12** |
| *MSC1* | **3.24** |  |  | *PUT1* | **2.12** |
| *ECM4* | **3.05** |  |  | *UBC5* | **2.11** |
| *ARN2* | **2.99** |  |  | *PTP2* | **2.11** |
| *FMP45* | **2.97** |  |  | *BIO4* | **2.10** |
| *GLC3* | **2.97** |  |  | *CUP2* | **2.08** |
| *PIR3* | **2.96** |  |  | *EMP46* | **2.08** |
| *ATG8* | **2.95** |  |  | *PDR5* | **2.06** |
| *CIT2* | **2.94** |  |  | *HSP150* | **2.05** |
| *PCA1* | **2.81** |  |  | *FIT1* | **2.05** |
| *GIP2* | **2.78** |  |  | *FMP23* | **2.05** |
| *YPS3* | **2.76** |  |  | *YER067w* | **2.04** |
| *TFS1* | **2.76** |  |  | *DUR1,2* | **2.04** |
| *HBT1* | **2.73** |  |  | *TIS11* | **2.04** |
| *SLT2* | **2.73** |  |  | *ADR1* | **2.03** |
| *GCY1* | **2.71** |  |  | *PDE1* | **2.03** |
| *FIT2* | **2.68** |  |  | *YNL200c* | **2.02** |
| *GSC2* | **2.67** |  |  |  |  |
| *RTS3* | **2.62** |  |  |  |  |
| *IDP2* | **2.50** |  |  |  |  |
| *TSL1* | **2.50** |  |  |  |  |
| *CRH1* | **2.46** |  |  |  |  |
| *HMX1* | **2.46** |  |  |  |  |
| *GDB1* | **2.44** |  |  |  |  |
| *JAC1* | **2.43** |  |  |  |  |
