# Supplementary material for: Ptc6 Is Required for Proper Rapamycin-Induced Down-Regulation of the Genes Coding for Ribosomal and rRNA Processing Proteins in S. cerevisiae
Source: PLoS One. 2013 May 21;8(5):e64470. doi: 10.1371/journal.pone.0064470 (PMC3660562; doi:10.1371/journal.pone.0064470)
Supplement: Table S3 — Genes down-regulated in ptc1 ptc6 cells. (DOCX) [file pone.0064470.s007.docx]

**Table S3. Genes down-regulated in *ptc1 ptc6* cells.**

|  | | | | |  |  |  |  |
| --- | --- | --- | --- | --- | --- | --- | --- | --- |
|  | **GENE** | **-Fold decrease** |  |  |  |  |  |  |
|  | *PTC6* | **0.06** |  |  |  |  |  |  |
|  | *PTC1* | **0.17** |  |  |  |  |  |  |
|  | *YAR068w* | **0.27** |  |  |  |  |  |  |
|  | *LEU1* | **0.35** |  |  |  |  |  |  |
|  | *CUP1-1* | **0.45** |  |  |  |  |  |  |
|  | *OAC1* | **0.46** |  |  |  |  |  |  |
|  | *RPS27b* | **0.46** |  |  |  |  |  |  |
|  | *CYB5* | **0.47** |  |  |  |  |  |  |
|  | *ALD6* | **0.47** |  |  |  |  |  |  |
|  | *PHO12* | **0.47** |  |  |  |  |  |  |
|  | *PHO11* | **0.48** |  |  |  |  |  |  |
|  | *GFD2* | **0.48** |  |  |  |  |  |  |
|  | *RPS17a* | **0.49** |  |  |  |  |  |  |
|  | *RPL25* | **0.49** |  |  |  |  |  |  |
